# Functional annotation and comparative genomics analysis of *Balamuthia mandrillaris* reveals potential virulence-related genes

**DOI:** 10.1038/s41598-023-41657-6

**Published:** 2023-08-31

**Authors:** Alejandro Otero-Ruiz, Libia Zulema Rodriguez-Anaya, Fernando Lares-Villa, Luis Fernando Lozano Aguirre Beltrán, Luis Fernando Lares-Jiménez, Jose Reyes Gonzalez-Galaviz, Abraham Cruz-Mendívil

**Affiliations:** 1 Programa de Doctorado en Ciencias Especialidad en Biotecnología, Departamento de Biotecnología y Ciencias Alimentarias, Instituto Tecnológico de Sonora, 85000 Ciudad Obregón, Sonora Mexico; 2 CONAHCYT-Instituto Tecnológico de Sonora, 85000 Ciudad Obregón, Sonora Mexico; 3 Departamento de Ciencias Agronómicas y Veterinarias, Instituto Tecnológico de Sonora, 85000 Ciudad Obregón, Sonora Mexico; 4 Unidad de Análisis Bioinformáticos, Centro de Ciencias Genómicas de la Universidad Nacional Autónoma de México (UNAM), 62210 Cuernavaca, Morelos Mexico; 5 CONAHCYT-Instituto Politécnico Nacional, CIIDIR Unidad Sinaloa, 81101 Guasave, Sinaloa Mexico

**Keywords:** Bioinformatics, Whole genome amplification, Comparative genomics, DNA sequencing, Next-generation sequencing

## Abstract

*Balamuthia mandrillaris* is a pathogenic protozoan that causes a rare but almost always fatal infection of the central nervous system and, in some cases, cutaneous lesions. Currently, the genomic data for this free-living amoeba include the description of several complete mitochondrial genomes. In contrast, two complete genomes with draft quality are available in GenBank, but none of these have a functional annotation. In the present study, the complete genome of *B. mandrillaris* isolated from a freshwater artificial lagoon was sequenced and assembled, obtaining an assembled genome with better assembly quality parameter values than the currently available genomes. Afterward, the genome mentioned earlier, along with strains V039 and 2046, were subjected to functional annotation. Finally, comparative genomics analysis was performed, and it was found that homologous genes in the core genome potentially involved in the virulence of *Acanthamoeba* spp. and *Trypanosoma cruzi*. Moreover, eleven of fifteen genes were identified in the three strains described as potential target genes to develop new treatment approaches for *B. mandrillaris* infections. These results describe proteins in this protozoan's complete genome and help prioritize which target genes could be used to develop new treatments.

## Introduction

*Balamuthia mandrillaris* is a free-living amoeba (FLA) widely distributed in the environment of the warmer countries^1^. It is the causal agent of a chronic infection called *Balamuthia* amoebic encephalitis (BAE), and in some cases, skin lesions precede infection of the central nervous system (CNS)^2^. Additionally, it has been reported that this infection affects immunocompetent and immunocompromised people, and currently, more than 200 cases have been reported worldwide with a mortality rate > 90%, with most of these cases occurring in the United States and South America^3^. This high mortality rate is primarily due to the difficulty of obtaining an early diagnosis (when the disease may be manageable), coupled with the lack of specific drugs for *B. mandrillaris* infections; the current treatment consists of a combination of antimicrobials selected mostly empirically, resulting in few cases of survival at present^4^. For this reason, it is necessary to implement techniques that involve omic sciences in the study of this FLA to identify known protein domains for advancing to functional annotation and provide tools for the knowledge of the pathogenomics of this protozoan^5^.

Currently, the genomic information of this microorganism is scarce, and only the mitochondrial genomes of different isolates have been annotated, with lengths ranging from 39.8 to 42.8 Kb, 2 ribosomal RNAs (rRNAs), 13 to 18 transfer RNAs (tRNAs), and 33 to 38 protein-coding sequences^4, 6^. In a recent study, the *B. mandrillaris* transcriptome was analyzed, approximately 40% of the predicted proteins were functionally annotated, and 15 target genes for new treatment approaches for *B. mandrillaris* infections were identified^7^. However, there is no complete annotated genome of this FLA in GenBank, and only two draft quality genomes are available for strains 2046 and V039, which vary in size from 44 to 68 Mb, respectively^8, 9^.

Regarding other microorganisms of medical relevance, studies that combine functional annotation and comparative genomics have been reported to identify genes related to antibiotic resistance, virulence factors, transcriptional regulators, motility, and others^10–13^. Pangenome analysis of FLA revealed unique genes in pathogenic *Acanthamoeba* and *Naegleria fowleri* species. For *Acanthamoeba*, genes involved in virulence were reported as metalloproteases, laminin-binding proteins, and heat shock proteins^14^. For *Naegleria fowleri*, genes related to autophagy, cytoskeletal and membrane dynamics, motility, secretory products, response to stress, and posttranslational modifications were identified^15^.

The scarcity of genomic information has hampered the development of new compounds against *B. mandrillaris*. Therefore, combining functional annotation and comparative genomics of this pathogenic protozoan could help understand the genomic biology and identify conserved genes among different strains^7, 16, 17^. This study presents the annotation of the draft genomes of *B. mandrillaris* in GenBank, the genome assembly and annotation of a strain isolated in an artificial lagoon, and comparative genomics of the different strains.

## Materials and methods

### Maintenance of *B. mandrillaris*

The *B. mandrillaris* strain ITSON01 was isolated in 2014 from an artificial lagoon in Ciudad Obregon, Mexico^18^. Trophozoites were cultured axenically with *Balamuthia mandrillaris* ITSON medium in 75 cm^2^ ventilated cell culture bottles at 37 °C^19^. Trophozoites were harvested for DNA extraction.

### DNA extraction and sequencing

Trophozoites were resuspended in phosphate-buffered saline (PBS, pH 7.4) using 6 cell culture bottles of 75 cm^2^ (approximately 10.8 × 10^6^ cells). DNA extraction was performed using the Wizard SV Genomic DNA Purification System (Promega, Madison, WI) according to the manufacturer's instructions, obtaining 0.46 µg total DNA. The libraries were then sequenced at the genomic services laboratory (LABSERGEN, Irapuato, Gto) using the Illumina NextSeq platform with 150 bp paired-end reads, yielding approximately 50 million reads.

Furthermore, DNA extraction for sequencing with Oxford Nanopore Technologies (ONT) was performed by harvesting trophozoites with PBS washes as previously described, and a total of 20 cell culture bottles of 75 cm^2^ (approximately 36 × 10^6^ cells) were used. Subsequently, the extraction was performed using the Wizard HMW DNA Extraction Kit (Promega, Madison, WI) according to the manufacturer's instructions, obtaining 6.89 µg total DNA. The libraries were then sequenced at the company Health GeneTech (HGT, Taoyuan, TW) with the ONT gridION platform, yielding a total of approximately 3 million reads (8.5 Gb of total bases). The length distribution of the raw long ONT reads was plotted using NanoPlot v1.41.0^20^.

### Assembly and annotation of the mitochondrial genome of *B. mandrillaris* strain ITSON01

The mitochondrial genome (mtDNA) assembly of *B. mandrillaris* was performed using short reads only (Illumina). Raw reads were filtered with default parameters for quality and minimum length using Trim Galore v0.6.4^21^ and de novo assembled using SPAdes v3.13^22^. Once the assembly was obtained, to identify the mtDNA, the synteny was defined by alignment with 8 complete mtDNA (GenBank accession number: KT175738, KT175739, KT030672, KT175740, KT030671, KT030673, KT175741, and KT030670) using Mauve^23^. Subsequently, the aforementioned mitochondrial genome was isolated, and annotation of tRNA and protein coding genes (CDS) was performed with GeSeq^24^, whereas the rRNAs were identified with barrnap v0.9^25^. Finally, the rRNAs were appended to the GeSeq output file by manual curation using Artemis^26^. A comparison of ITSON01 mtDNA was performed with the 8 genomes mentioned above and one more recently published genome (GenBank accession number: OM994889) using the CGview Comparison Tool (CCT)^27^.

### Hybrid genome assembly of *B. mandrillaris* strain ITSON01 and genome reassembly of *B. mandrillaris* strain 2046

The long ONT raw reads were subjected to adapter removal with Porechop v0.2.4^28^ and quality filtering with Filtlong v0.2.0^29^, which eliminated reads with lengths less than 1 kb and ignored the phred quality values of the ONT reads, instead judging the quality using K-mer matches with the short Illumina reads^30–32^. The hybrid assembly was performed with default parameters using MaSuRCA v4.0.5^33^, taking the raw short Illumina reads and filtered long ONT reads as input. The genome of *B. mandrillaris* strain 2046 was also reassembled using the reads available (GenBank accession numbers: SRR8980854, SRR8980855, and SRR8980856) for this strain and assembled with MaSuRCA v4.0.9^8^.

### RNA extraction and sequencing from *B. mandrillaris* ITSON01

RNA extraction for poly(A) and total RNA sequencing was performed using a RNeasy Minikit (QIAGEN, Hilden, Germany) according to the manufacturer’s instructions (only extraction temperature at 4 °C and centrifugation time were modified from 15 s to 1 min). RNA integrity was determined by the 2100 Bioanalyzer System (Agilent, Santa Clara, CA). The poly(A) libraries were sequenced at LC Sciences (Houston, TX) on an Illumina NovaSeq platform with 150 bp paired-end reads, yielding approximately 40 million reads per library. Moreover, the total RNA library was sequenced on the same company and platform, with 150 bp paired-end reads and a sequencing depth of approximately 200 million reads.

### Annotation of proteins and noncoding genes in *B. mandrillaris* genomes

The complete genomes of the three strains were subjected to gene prediction and annotation using Funannotate v1.8.14^34^, taking as input the genome assembly of each strain and RNA-seq reads. Pannzer2^35^ was used when Funannotate was unable to assign a functional description with the following parameter settings: minimum query coverage 0.4 or minimum sbjct coverage 0.4 and minimum alignment length 50, obtaining the proteins and tRNA annotated^36, 37^. Subsequently, the rRNAs (28S, 18S, and 5S) and long noncoding RNAs (lncRNAs) were predicted using the genome assembly of each strain as input with StructRNAfinder^38^, obtaining the location in the assembly and structure of these nonprotein-coding RNAs as output. Once these nonprotein-coding genes were obtained, manual curation was performed using Geneious Prime v2023.0.4^39^. Finally, gene ontology (GO) terms were extracted from the Funannotate results and visualized on WEGO 2.0^40^.

### Comparative genomics

Comparative genomics analysis was performed using annotated protein sequences of the three *B. mandrillaris* strains (ITSON01, CDC-V039 and 2046) with default parameters using GET_HOMOLOGUES^41, 42^, obtaining the sequence cluster belonging to the pan/core genome as an output file.

## Results

### Filtering on Illumina and ONT DNA-Seq reads

**Figure 1:**
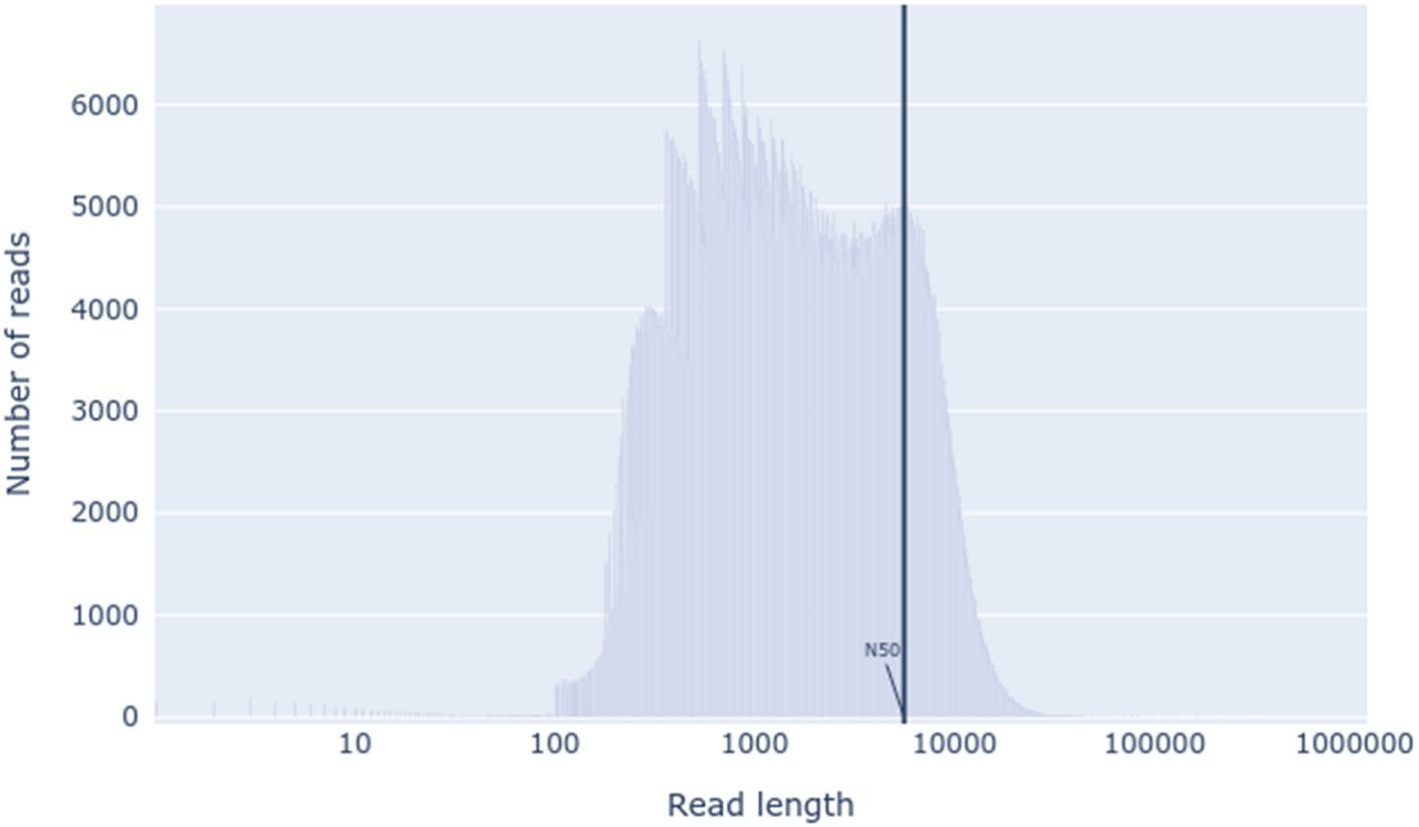
Length distribution of the raw long ONT reads.

After filtering with Trim Galore, approximately 0.18% of the Illumina reads were removed, reducing from 50,109,114 to 50,020,496 reads. The low number of reads removed is due to the high quality and depth coverage of the sequences from the Illumina platforms^43^. In the ONT reads, after adapter trimming with Porechop and quality filtering with the Illumina reads as a reference with Filtlong, approximately 43.74% of the reads were removed, reducing from 3,095,072 to 1,741,403 reads, possibly due to the large number of reads smaller than 1 kb (Fig. 1). However, most of the total bases were retained, eliminating approximately 11% after the filtering process.

### Assembly and annotation of the mitochondrial genome of *B. mandrillaris* ITSON01

**Figure 2:**
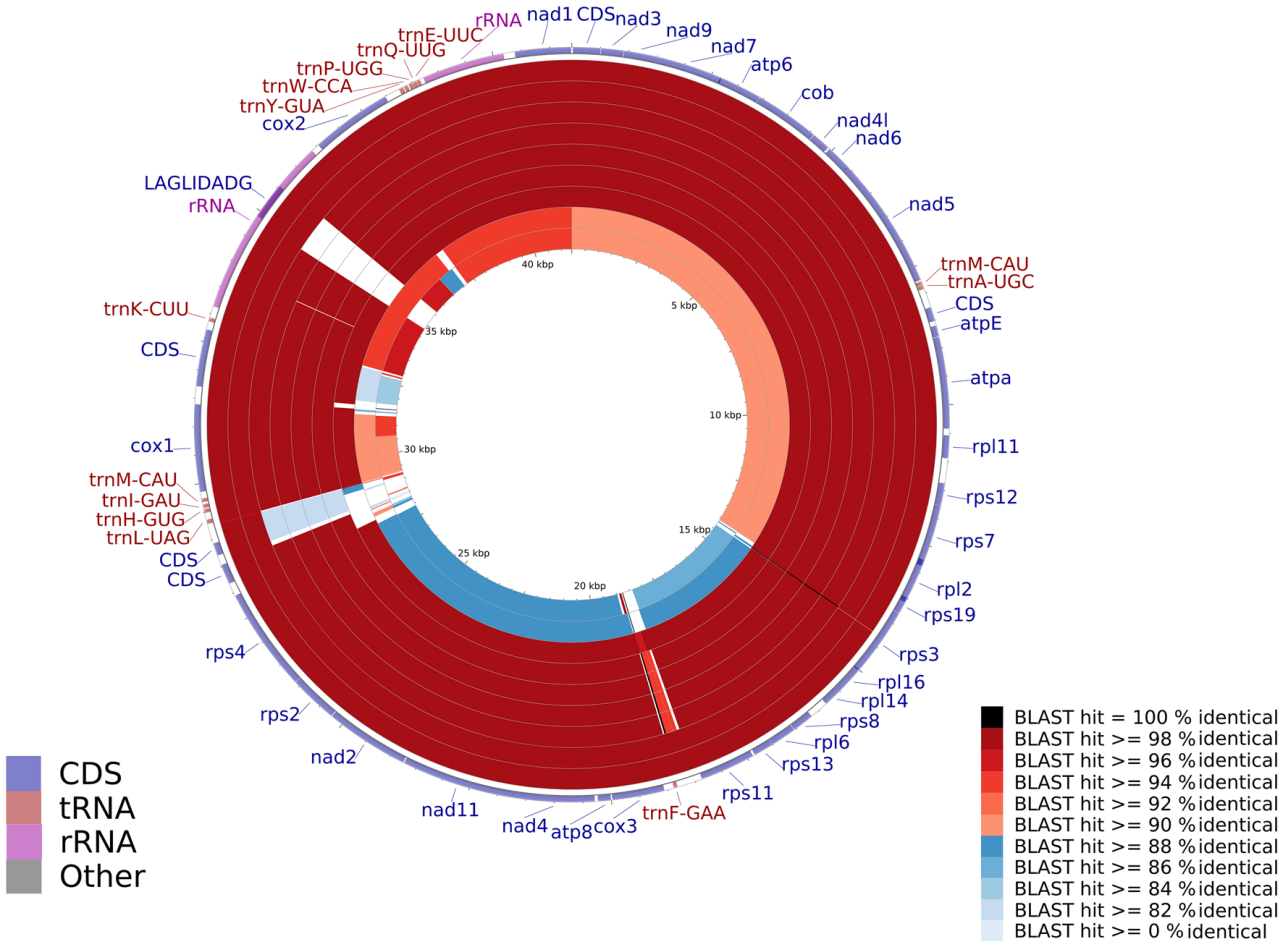
Comparison of *B. mandrillaris* mtDNA against mtDNA from different strains (from edge to center, the strains are V118, GAM19, RP5, 2046, OK1, SAM, V039, V451, and KM-20).

After assembly and annotation, mtDNA was obtained with a length of 41,385 bp, 13 tRNA, 37 CDS, and 2 rRNA subunits. The mtDNA of *B. mandrillaris* strain ITSON01 was compared against some mtDNA of different strains available in GenBank, showing that most have an identical percentage > 98% except for strains V451 and KM-20 (Fig. 2).

### Assembly of the *B. mandrillaris* strain ITSON01 genome and reassembly of the *B. mandrillaris* strain 2046 genome

After the hybrid assembly of *B. mandrillaris* strain ITSON01, a genome of approximately 65 Mb was obtained with better assembly quality values, such as the number of contigs, N50, L50, and low amount of "N" in the genome, than those currently available. Instead, reassembling the genome of *B. mandrillaris* strain 2046 resulted in a less fragmented genome, larger genome size, and lower "N" than the current genome of this strain (Table 1)^8, 9^. This reassembly was used for functional and comparative genomic annotation.

**Table 1:** Comparison of assembled genomes of different strains of *B. mandrillaris***.**

*Balamuthia mandrillaris* assembled genomes				
Parameters	2046	V039	ITSON01	2046 reassembly
Scaffolds number	14,699	1605	841	10,760
Longest contig	220,212	914,981	835,953	76,855
Assembly size	44,270,879	67,656,513	65,200,415	57,592,625
N50	26,144	93,953	160,199	9882
L50	369	160	123	1644
GC (%)	46.83	46.77	46.41	46.78
# N's	1,379,769	25	0	35,681

### Functional annotation of *B. mandrillaris* genomes

For the ITSON01 strain, 67% of its genes were annotated as proteins with functional descriptions, 31% as proteins without functional descriptions (hypothetical proteins), and 2% as noncoding genes (rRNA and tRNA). In the case of the V039 strain, 63% of its genes were identified as proteins with functional descriptions, 35% as hypothetical proteins, and 2% as rRNA and tRNA. Finally, for the 2046 strain, 63% of its genes were described as proteins with functional descriptions, 35% as hypothetical proteins, and 2% as tRNA.

It should be noted that in the case of the 2046 strain, complete ribosomal RNAs could not be annotated due to the high fragmentation of the genome. A detailed summary of the annotation results for each strain of *B. mandrillaris* is presented in Table 2. Regarding lncRNAs, in the ITSON01 strain, two were annotated as MESTIT1, one as NPPA-AS1, and one as TCL6, whereas in the V039 strain, three were annotated as CDKN2B-AS, NPPA-AS1, and Six3os1.

**Table 2:** Summary of functional annotations of the three strains of *B. mandrillaris*.

Species	Strain	Protein-coding genes			Non-protein-coding genes				
		Proteins with a functional description	Proteins without functional description	Total proteins	tRNA	28S-rRNA	18S-rRNA	5S-rRNA	Total ncRNA
*Balamuthia mandrillaris*	ITSON01	20,457	9510	29,967	441	15	30	42	528
	V039	18,638	10,283	28,921	427	53	27	38	545
	2046	19,100	10,413	29,513	560	N/A	N/A	N/A	560

*N/A* not annotated.

**Figure 3:**
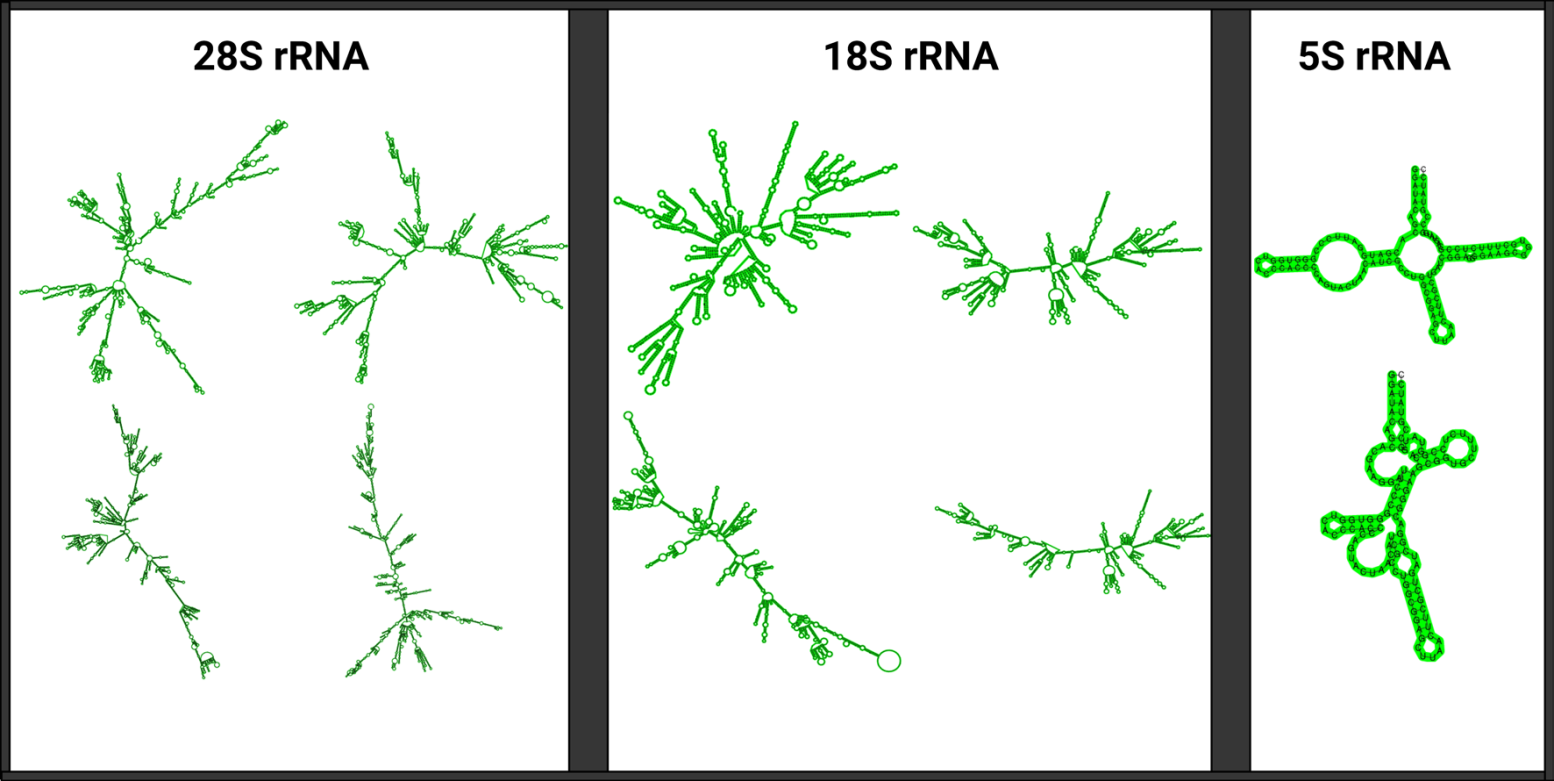
rRNA structures obtained with StructRNAfinder (created with BioRender.com).

Regarding the length distribution of the rRNA structures, in the ITSON01 strain, the large subunit (LSU) varied from 3625 to 5239 bp, and the small subunit (SSU) varied from 2017 to 2022 bp. In the V039 strain, the LSU varied from 3487 to 3853 bp, and the SSU varied from 2010 to 2028 bp. Additionally, the length of 5S rRNA was 119 bp in both strains. Some examples of structures of such rRNA obtained with StructRNAfinder are presented below (Fig. 3).

**Figure 4:**
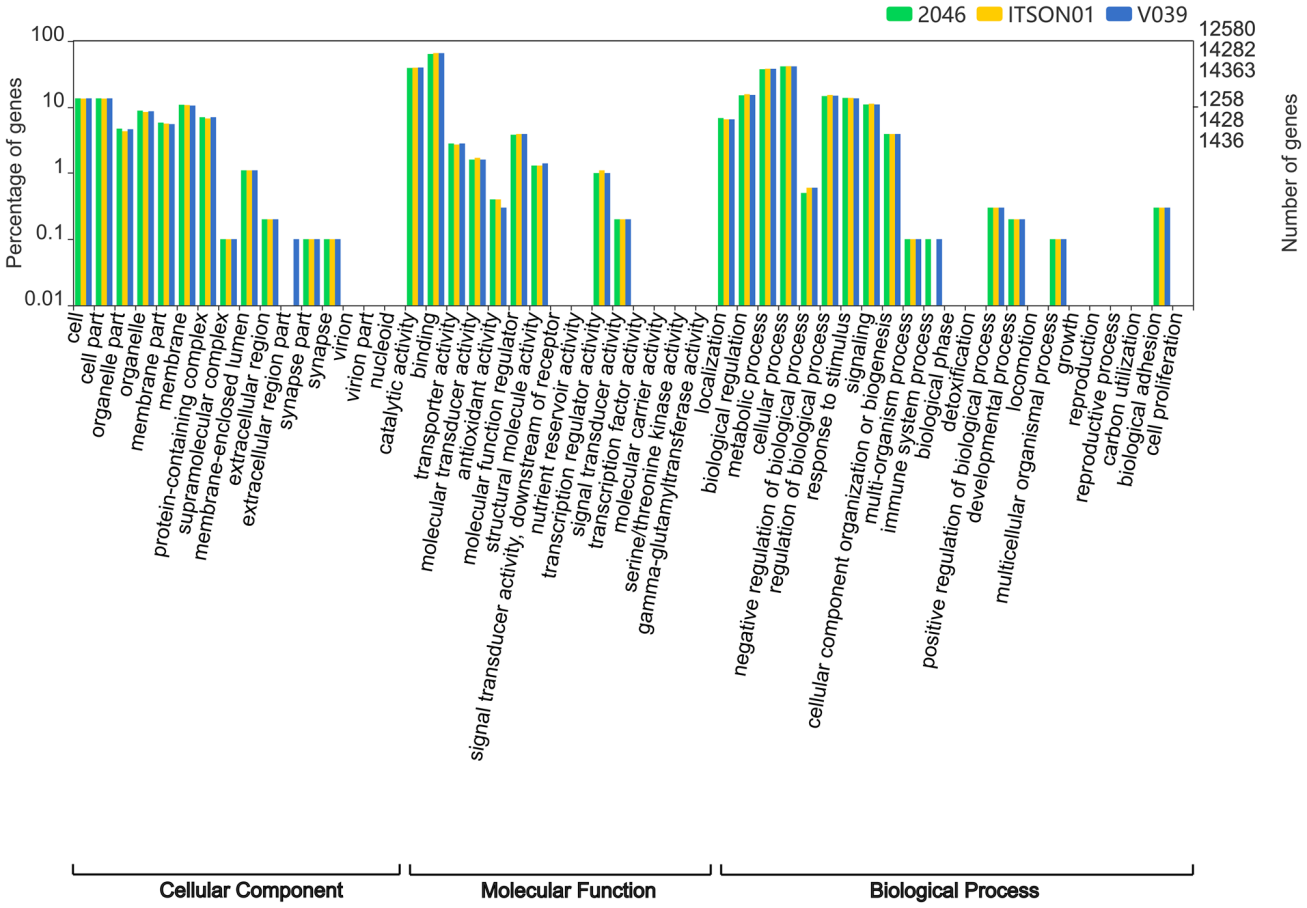
Level 2 GO annotations, proteins of *B. mandrillaris* 2046 (green), *B. mandrillaris* ITSON01 (yellow) and *B. mandrillaris* V039 (blue), percentages of genes and total number of genes are log scale (10).

The GO term annotation comparison revealed a similar profile for the 3 strains, except for some smaller gene families that represented less than 0.1% of the genes (Fig. 4). This analysis also revealed that the GO terms with the highest representation in the biological process category were "cellular process" (GO: 0009987) and "metabolic process" (GO: 0008152); for the cellular component category, they were "cell" (GO: 0005623) and "cell part" (GO: 0044464); and finally, for the molecular function category, they were "catalytic activity" (GO: 0003824) and "binding" (GO: 0005488).

### Comparative genomics

**Figure 5:**
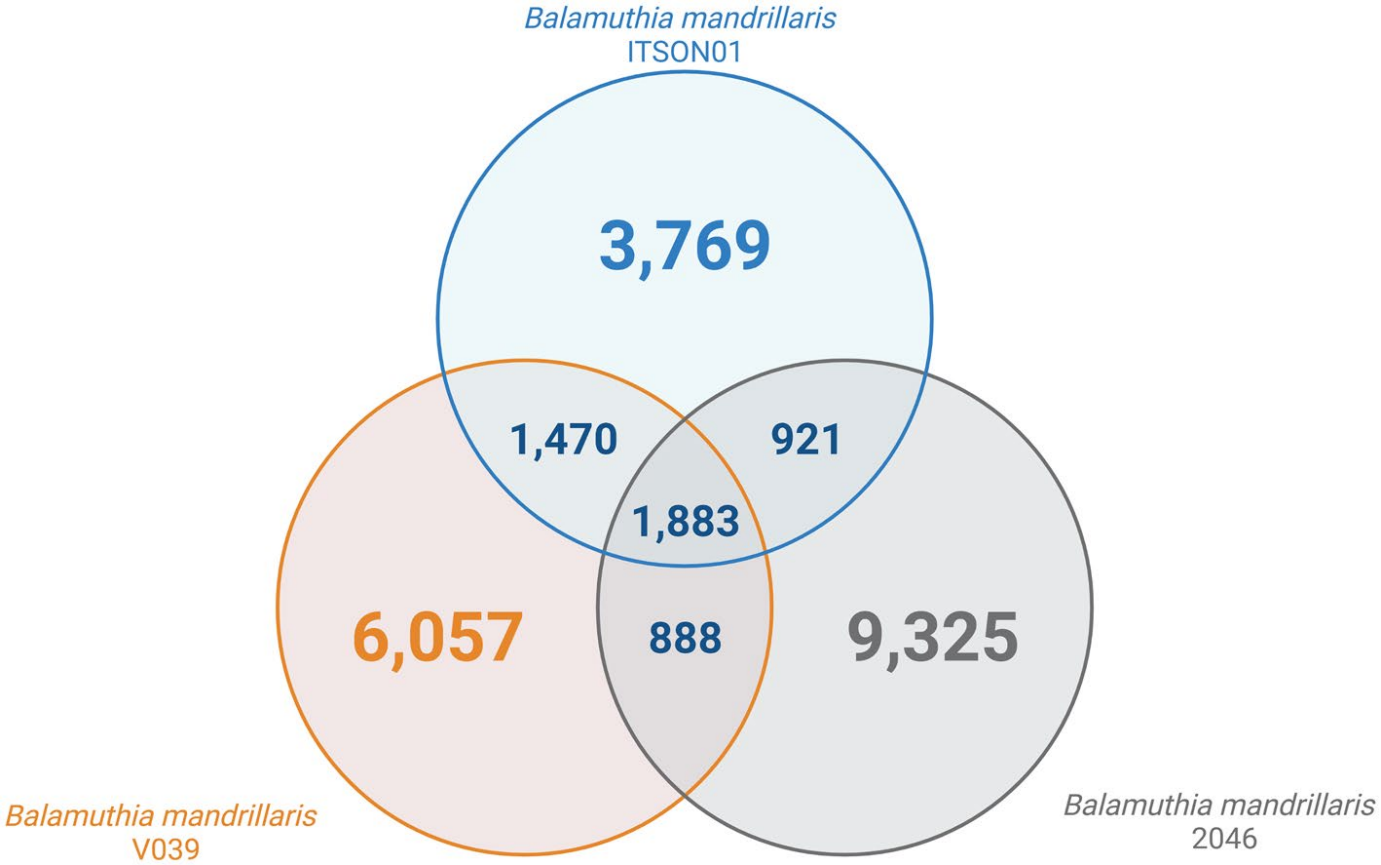
Venn diagram of overlap among orthologous groups in the proteomes of different strains of *B. mandrillaris*.

The results of the comparative genomics analysis were expressed in a Venn diagram (Fig. 5), which shows the overlap between orthologous groups of the different strains of *B. mandrillaris*. It should be noted that the orthologous gene clusters of the core genome represent approximately 6% of the proteins of each strain. At the same time, the numbers of unique protein genes, including the paralogs of each strain, were 4123 (13.8% of the total proteins), 6357 (22% of the total proteins), and 9732 (33% of the total proteins) for the ITSON01, V039, and 2046 strains, respectively.

## Discussion

Previous studies have described the different variations in the mitochondrial genomes of *B. mandrillaris*, one of which is the location of an open reading frame (ORF) endonuclease containing the sequence LAGLIDADG; in the case of the V039 strain, this is not present in the genome. In the 2046, OK1, RP-5, SAM and KM-20 strains, this sequence disrupts the cox1 gene, whereas in the V451, GAM-19, V188 and ITSON01 strains, it is inserted in the 23S ribosomal gene. Although more mitochondrial genomes are required for possible genotyping of *B. mandrillaris*, the contribution of the mitochondrial genome of the ITSON01 strain could help to achieve this in the future^5^.

The better assembly metrics of the nuclear genome observed in the ITSON01 strain compared to previous works are mainly due to the use of both short and long reads (Illumina and ONT), as well as the use of MaSuRCA software, which was designed for the assembly of large genomes and has been characterized for obtaining the best hybrid assembly quality parameters in various eukaryotic genomes^44–46^. In contrast, the genome reassembly of the 2046 strain considerably improved with respect to the original assembly due to the use of MaSuRCA; this is possibly because this program has a record of obtaining better N50 length values than SPAdes and therefore having lower fragmentation in the assembled genomes using this program^47^. Furthermore, the reassembly was not improved compared to the genomes of the other strains (ITSON01 and V039) because only short reads were used; it is known that the use of only short reads in eukaryotic genome assemblies results in higher fragmentation (more scaffolds)^48^.

A larger number of genes with annotated functional descriptions were obtained due to the use of two programs, Funannotate and Pannzer2. Funannotate uses various curated databases to perform functional annotations, such as PFAM, InterPro, MEROPS, and CAZy, and to determine gene names and descriptions using EggNOG and UniProtKb/SwissProt, the latter consisting of manually reviewed high-quality protein sequences (approximately 0.5 million sequences). Additionally, Pannzer2 uses two UniProtKb databases, SwissProt and TrEMBL, the latter consisting of computationally reviewed high-quality protein sequences (approximately 208 million sequences). Therefore, the large number of sequences consulted was of great help for the functional homology annotation of the *B. mandrillaris* genome^49–51^.

According to the Venn diagram, the 2046 strain has a higher number of unique proteins than the other strains, which could be because genome fragmentation can be correlated with the duplication of specific sequences within the assembled genome^52^. In the cloud genome of strain ITSON01, several paralogous genes coding for protease (without group classification) and Vps9 domain-containing protein were identified, the latter of which has been found in other FLAs of medical importance, such as *Naegleria fowleri*^5^. Meanwhile, within the orthologous groups of the core genome of the three strains, homologous genes potentially involved in host invasion of pathogenic *Acanthamoeba* species were identified, which are SH3 domain-containing protein, filamin repeat domain-containing protein, myosin II light chain 1, myosin IA, heat shock protein 20, superoxide dismutase, metacaspase, and RAP7; the first 4 genes are related to the cytoskeleton, ability to tolerate high temperatures, defense against reactive oxygen species, phagocytosis process and endosomal delivery after phagocytosis (dominates energy production and cell growth), respectively (Supplementary Table S1)^14, 53^. Furthermore, homology was also found for a cysteine protease called cruzipain from the pathogenic protozoan *Trypanosoma cruzi*, which plays important functions in this protozoan, such as evasion of the immune response, differentiation, metabolism and invasion of host cells^54^. Therefore, it is likely that these aforementioned genes participate in similar molecular pathways and therefore have the same molecular functions in *B. mandrillaris*.

In other ways, we observed that within the three strains, a total of 11 of the previously published 15 sequences responsible for encoding target proteins to development of new treatments for *B. mandrillaris* infections were identified, which are methionyl-tRNA synthetase, xylose isomerase, heat shock protein 90, lanosterol 14-alpha demethylase, histone deacetylase, 3-hydroxy-3 methylglutaryl coenzyme A reductase, two types of DNA topoisomerase, calcium ATPase, glucokinase, and exportin-1^7^. Additionally, coding sequences for enzymes that facilitate destruction and migration through the host, such as metalloproteinases, phospholipase A_2,_ and phospholipase D, were found in all 3 strains^4^.

In the present study, homology was found with *Acanthamoeba castellanii* in the core genome of a gene encoding a serine carboxypeptidase. This type of enzyme has been described in silico as a pharmacological target in infections generated by *N. fowleri* because it is related to the virulence of this protozoan, as proven by genomic and transcriptomic studies. In their conclusion, they suggested that this enzyme has a ligand binding site suitable for design based on the structure of specific inhibitors, postulating it as a reliable target for treating primary amoebic meningoencephalitis (PAM) with drugs specifically aimed at blocking proliferation by inhibiting molecular function^55^.

Regarding the shell genome (ITSON01-V039), an extracellular protein aminopeptidase family M20/M25/M40 was determined to be homologous with the same pathogenic species of *Acanthamoeba* mentioned. This enzyme was shown to be involved in the *Acanthamoeba* pathogenesis process by pretreatment of proteins secreted by this FLA with leucine aminopeptidase inhibitor or specific antibiotic against the enzyme mentioned above, and a reduction in cell-based assay damage was observed^56^. In contrast to the other pathogenic species of FLA, nonpathogenic species of the genus *Balamuthia* have not yet been described. A new species of this genus has recently been reported (*Balamuthia spinosa*); however, it has not yet been described whether it is pathogenic in humans^57^. Therefore, differential expression analysis is still lacking to determine which proteins are involved in the pathogenesis of *B. mandrillaris*, but based on the evidence presented, the proteins, as mentioned earlier, could be related to the mechanisms employed by this pathogenic protozoan.

Regarding treatment development against *B. mandrillaris*, in a recent study, it was determined that formulations composed of azole (fluconazole and itraconazole) and 5-nitroimidazole (metronidazole) had a considerable antiparasitic effect against *N. fowleri* and *B. mandrillaris* amoebae, showing limited cytotoxic damage in human cells and reduction of host cell death caused by the pathogen^58^. In the present study, we found homology with bacteria and archaea (*Heimdallarchaeota*) in the core genome and shell (ITSON01-V039) genes coding for nitroreductases. These enzymes are important for the effectiveness of antimicrobials such as metronidazole, which requires a reduction in its nitro group to show antimicrobial effects^59^. Homology was also found with the FLA *A. castellanii* in the core genome genes coding for lanosterol 14-alpha demethylase, which has been described as a target in treatment with azoles^60^.

Another interesting finding to highlight is the identification of lncRNAs in the genome of *B. mandrillaris*. This type of noncoding RNA has been shown to be significant for its participation in development and physiological processes through its regulation of gene expression^61^. One of the lncRNAs shared between the ITSON01 and V039 strains was NPPA-AS1. In previous studies, an increase in this type of lncRNA was observed in HCT-8 cells infected with the pathogenic protozoan *Cryptosporidium parvum,* suggesting that this lncRNA, among others, could be involved in infection with this microorganism^62^. Regarding the other lncRNAs identified, no function related to infectious diseases is yet known. One aspect to note is that viral lncRNAs have also been attributed the ability to not induce an immune response compared to viral proteins, suggesting that viruses could use them as another strategy to invade their hosts^61^. Therefore, it is a desirable study area for pathogenic amoebas and other microorganisms capable of producing infections.

## Conclusion

In the present study, annotation of the nuclear and mitochondrial genomes of *B. mandrillaris* was achieved, obtaining valuable information about possible genes involved in the pathogenicity of this protozoan through homologs with other pathogenic protozoan species. However, studies supported in functional genomics to determine genes related to the virulence of this FLA are still lacking. In addition, the comparative genomics of different strains performed in this study helped to identify the homology between strains of target genes for possible treatment against *B. mandrillaris* infections, which could help in prioritizing the development of treatments for those target sequences presented.

### Supplementary Information

Supplementary Table S1.

## Data Availability

The datasets presented in this study can be found in online repositories. The repository names and accession numbers can be found under the BioProject: PRJNA975899 (https://dataview.ncbi.nlm.nih.gov/object/PRJNA975899?reviewer=slsojv2t5rc6q14qgbarhgnlp0).
